# Clonality and Resistome Analysis of KPC-Producing *Klebsiella pneumoniae* Strain Isolated in Korea Using Whole Genome Sequencing

**DOI:** 10.1155/2014/352862

**Published:** 2014-07-03

**Authors:** Yangsoon Lee, Bong-Soo Kim, Jongsik Chun, Ji Hyun Yong, Yeong Seon Lee, Jung Sik Yoo, Dongeun Yong, Seong Geun Hong, Roshan D'Souza, Kenneth S. Thomson, Kyungwon Lee, Yunsop Chong

**Affiliations:** ^1^Department of Laboratory Medicine, Research Institute of Bacterial Resistance, Yonsei University College of Medicine, 50 Yonsei-ro, Seodaemun-gu, Seoul 120-752, Republic of Korea; ^2^Chunlab, Inc., Seoul National University, Republic of Korea; ^3^School of Biological Sciences, Seoul National University, Republic of Korea; ^4^Department of Science, University of British Columbia, Vancouver, Canada; ^5^Division of Antimicrobial Resistance, Korea National Institute of Health, Osong, Republic of Korea; ^6^Department of Laboratory Medicine, CHA Bundang Medical Center, CHA University, Seongnam, Republic of Korea; ^7^Department of Medical Microbiology and Immunology, Center for Research in Anti-Infectives and Biotechnology, Creighton University School of Medicine, Omaha, NE, USA

## Abstract

We analyzed the whole genome sequence and resistome of the outbreak *Klebsiella pneumoniae* strain MP14 and compared it with those of *K. pneumoniae* carbapenemase- (KPC-) producing isolates that showed high similarity in the NCBI genome database. A KPC-2-producing multidrug-resistant (MDR) *K. pneumoniae* clinical isolate was obtained from a patient admitted to a Korean hospital in 2011. The strain MP14 was resistant to all tested *β*-lactams including monobactam, amikacin, levofloxacin, and cotrimoxazole, but susceptible to tigecycline and colistin. Resistome analysis showed the presence of *β*-lactamase genes including *bla*_KPC-2_, *bla*_SHV-11_, *bla*_TEM-169_, and *bla*_OXA-9_. MP14 also possessed *aac(6*′-*)Ib*, *aadA2,* and *aph(3*′-*)Ia* as aminoglycoside resistance-encoding genes, *mph(A)* for macrolides, *oqxA* and *oqxB* for quinolone, *catA1* for phenicol, *sul1* for sulfonamide, and *dfrA12* for trimethoprim. Both SNP tree and cgMLST analysis showed the close relatedness with the KPC producers (KPNIH strains) isolated from an outbreak in the USA and colistin-resistant strains isolated in Italy. The plasmid-scaffold genes in plasmids pKpQil, pKpQil-IT, pKPN3, or pKPN-IT were identified in MP14, KPNIH, and Italian strains. The KPC-2-producing MDR *K. pneumoniae* ST258 stain isolated in Korea was highly clonally related with MDR *K. pneumoniae* strains from the USA and Italy. Global spread of KPC-producing *K. pneumoniae* is a worrying phenomenon.

## 1. Introduction

*Klebsiella pneumoniae* carbapenemase- (KPC-) producing* K. pneumoniae* has spread worldwide after the initial report in the USA [1] and has become a serious problem in nosocomial infections due to the associated high mortality, which can be as high as 50% [2–4]. KPC-2 is one of the most common carbapenemases in Enterobacteriaceae in the USA. Multilocus sequence typing (MLST) sequence type (ST) 258 is a common type among KPC-2-producing* K. pneumoniae* in various parts of the world [5–8]. In Korea, KPC-2-producing* K. pneumoniae* ST11 first was detected in 2010, and a second case of KPC-2-producing* K. pneumoniae* ST258 was also reported [9]. Subsequently, an outbreak of three cases of KPC-2-producing* K. pneumoniae *was detected in a teaching hospital in Korea [10].

Pulsed-field gel electrophoresis (PFGE) and MLST have been widely used to understand bacterial dissemination. However, to control the further spread of KPC-2-producing* K. pneumoniae,* a better understanding of their mode of transmission is required. With rapid technological advances, whole genome sequencing (WGS) using a massive parallel sequencer is now becoming a standard protocol in bacterial typing.

Here, we analyzed the whole genome sequence and resistome of the outbreak strain MP14 and compared it with those of KPC-2-producing isolates in the NCBI genome database (http://www.ncbi.nlm.nih.gov/genome) that showed high similarity to obtain insight into their mode of transfer.

## 2. Materials and Methods 

### 2.1. Bacterial Isolates and Antimicrobial Susceptibility Testing

The strain MP14 was isolated from the sputum sample of a 72-year-old man with pneumonia in a Korean hospital in 2011. He had no recent travel history. The species was identified by conventional methods and the VITEK 32 GN system (bioMérieux, Marcy l'Etoile, France). Antimicrobial susceptibility testing was performed using the VITEK II N211 system (bioMérieux).

### 2.2. Whole Genome Sequencing

Genome sequence of MP14 was obtained using a combination of Illumina Miseq (150 bp paired end) and Roche 454 (0.8 kb insert paired end) sequencing systems. A total of 3,445,050 paired reads were obtained from Miseq run (Q30 > 78%), and 199,522 reads were obtained from 454 sequencing systems. The sequences obtained from Miseq were assembled using the CLC genomic workbench (CLC Bio, Denmark), and the sequences from the 454 sequencing systems were assembled using GS De Novo Assembler 2.3 (Roche Diagnostics, Branford, CT). The length of minimum contig was 500 bp, and mismatch cost (2), insertion cost (3), deletion cost (3), length fraction (0.5), and similarity fraction (0.8) were used for assembly of Illumina reads in CLC genomic workbench. For assembly 454 sequence reads, the length of minimum overlap was 40 bp, and the identity of minimum overlap was 90%. Alignment identity score (2) and alignment difference score (−3) were used in GS De Novo Assembler. Hybrid assembly of strain MP14 sequences from Miseq and 454 was conducted using CodonCode Aligner (CodonCode Co., MA). The genes were identified with Glimmer (maximum overlap length was 50, minimum gene length was 110, and threshold score for calling gene was 30) [11], and annotations were provided by homology search against COG and SEED databases (database obtained at 2012-1-28) [12, 13]. The whole genomes of the strains sequenced in this study were compared with the reported genome sequences of* K. pneumoniae* isolates in the NCBI genome database.

### 2.3. Identification of Resistomes and Plasmids

Antimicrobial resistance genes and plasmid types were analyzed using ResFinder and PlasmidFinder, respectively, and using resources from the Center for Genomic Epidemiology (http://www.genomicepidemiology.org). ResFinder threshold of ID = 98% and PlasmidFinder threshold of ID = 95% were selected.

### 2.4. PFGE and Southern Blotting

Whole genomic DNA of isolates was digested with S1 nuclease (Invitrogen, Abingdon, UK) and PFGE was performed using a CHEF-DRII device (Bio-Rad, Hercules, CA) as described previously [14]. Gels with PFGE-separated fragments of DNA were blotted onto nylon membranes (Bio-Rad) and hybridized with probes specific for the *bla*_KPC-2_ gene using the DIG DNA Labeling and Detection Kit (Roche Diagnostics GmbH, Mannheim, Germany).

### 2.5. SNP Tree and cgMLST Analysis

The single nucleotide polymorphism (SNP) tree web server of the Center for Genomic Epidemiology (http://cge.cbs.dtu.dk) was used to generate SNPs tree. The minimum coverage for SNP calls was 10, the minimum distance between SNPs (prune) was 10 bp, and the minimum distance to the end of sequence from a reference sequence end was 0 bp. Genome sequence of* K. pneumonia* 342 (CP000964) was used as reference for SNP calls.

A core genome MLST (cgMLST) scheme was defined using the Ridom SeqSphere^+^ software (Ridom GmbH, Munster, Germany) with default settings [15]. The genome of the* K. pneumoniae* KCTC2242 served as reference genome and the following seven query genomes were used:* K. pneumoniae *(strains NC_018522.1, NC_011283.1, NC_022566.1, NC_016845.1, NC_022082.1, NC_009648.1, and NC_012731.1). Default settings included the removal of the shorter of two genes overlapping by more than four bases and of genes with an internal stop codon in more than 80% of all query genomes from the scheme [15].

## 3. Results

The strain MP14 was resistant to multiple antibiotics including ampicillin (MIC, ≥32 mg/L), ampicillin-sulbactam (≥32 mg/L), piperacillin-tazobactam (≥128 mg/L), cefoxitin (≥64 mg/L), ceftazidime (≥64 mg/L), cefotaxime (32 mg/L), cefepime (16 mg/L), aztreonam (≥64 mg/L), meropenem (≥16 mg/L), cotrimoxazole (≥32 mg/L), amikacin (≥64 mg/L), and levofloxacin (≥8 mg/L) but was susceptible to gentamicin (≤1 mg/L), tigecycline (2 mg/L), and colistin (0.5 mg/L).

**Figure 1:**
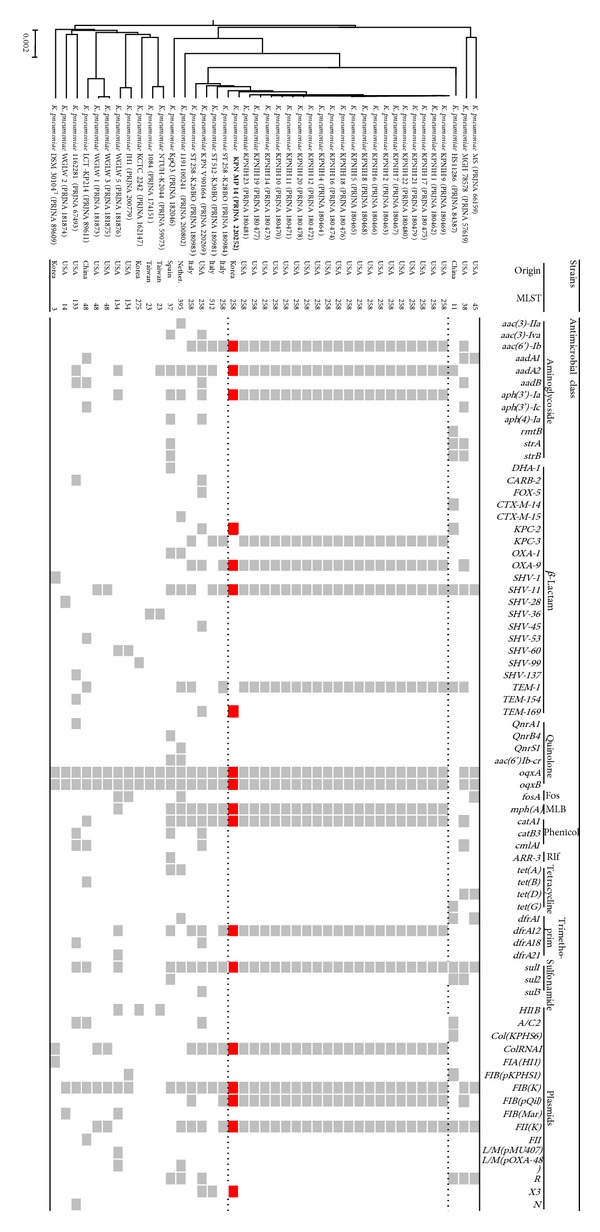
A genome tree and resistome analysis of strain MP14 and similar strains obtained from the NCBI database. The genome tree (left) was constructed using the UPGMA dendrogram based on average nucleotide identity values. The scale bar indicates the difference of ANI values among strains. In resistome analysis (right), boxes show the presence (gray and red) or absence (white) of the relevant genes for each of the isolates. Fos: fosfomycin; MLB: macrolide-lincosamide-streptogramin B; Rif: rifampicin.

**Figure 2:**
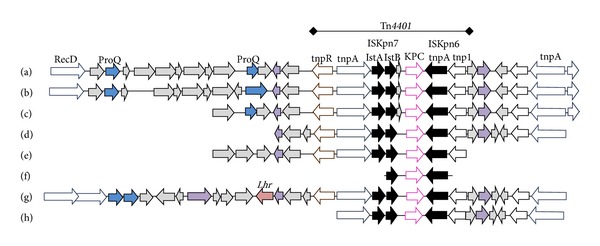
Comparison of genes identified in contigs containing the *bla*_KPC_ gene in strain MP14 (d) and representative strains showing high similarity: KPNIH14 (a), KPNIH19 (b), KPNIH23 (c), ST258-K28BO (e), ST512-K30BO (f), KPN V901664 (g), and ST258-K26BO (h).

A total of 186 contigs representing 5,661,622 bases (56.98% G+C ratio, N50 = 156,843) were obtained from hybrid assembled sequences of strain MP14 (183.76 × coverage). 5,708 CDS, 4 rRNA genes, and 77 tRNA genes were annotated for final contigs. A genome tree composed of strain MP14 and 40 related strains obtained from the NCBI database (date last accessed, 1 October 2013) was constructed using the UPGMA dendrogram based on average identity (ANI) value [16] computed by a BLAST algorithm (Figure 1). Strains in the NCBI database similar to MP14 were as follows: KPNIH strains from outbreak patients in the USA [17]; ST258-K28BO, ST512-K30BO, and ST258-K26BO isolated from patients in Italy [18]; and KPN V901664, a strain isolated in the early 2000s in the USA. The identity value between MP14 and these strains was over 99.8% in ANI analysis. All of those isolates carried *bla*_KPC-2_ or *bla*_KPC-3_ genes. All of these isolates were MLST ST258 except for one (ST512-K30BO). The* K. pneumoniae* HS11286 strain carrying the *bla*_KPC-2_ gene from China was ST11. A length of the Tn*4401* region harboring *bla*_KPC_ alleles was observed in some of the compared strains including KPNIH strains 14, 19, and 23, ST258-K28BO, ST512-K30BO, ST258-K26BO, and V901664 (Figure 2). Furthermore, the genetic environment around Tn*4401* was highly similar among the compared strains, suggesting the existence of a larger mobile element than Tn*4401* carrying the *bla*_KPC_ gene.

**Figure 3:**
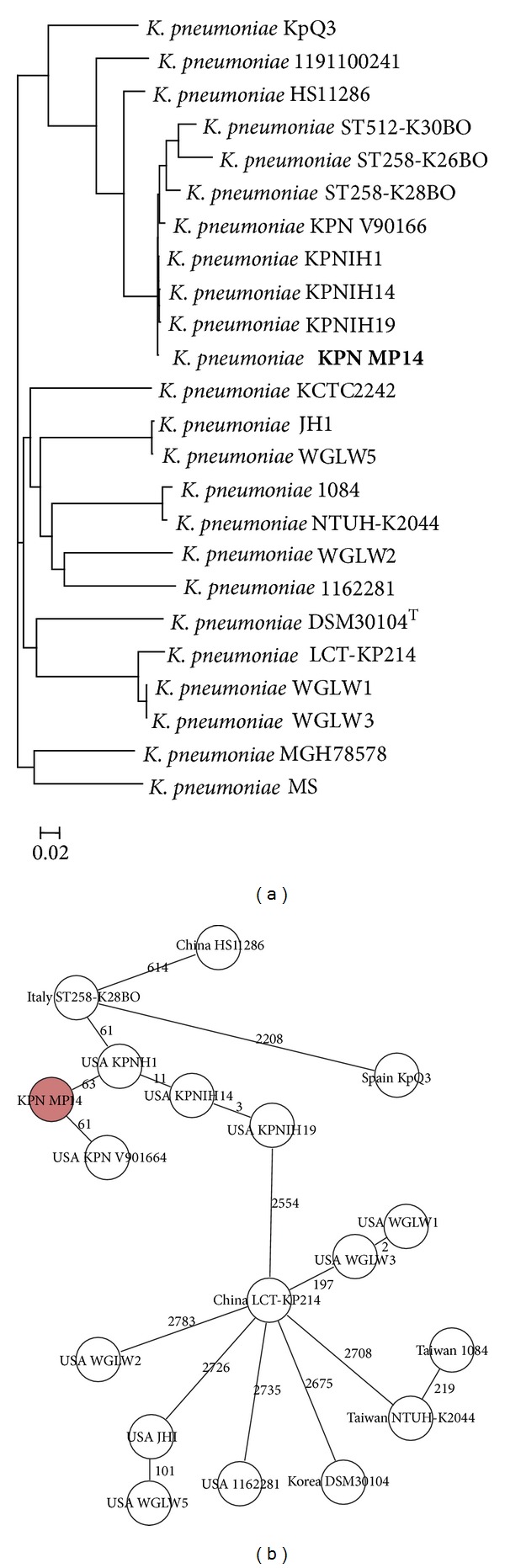
SNP tree (a) and cgMLST analysis (b) using whole genome sequences of the strain MP14 and similar strains obtained from the NCBI database.

*K. pneumonia *KPN MP14 was the most similar to KPNIH strains than other strains in SNP tree (Figure 3). Strain MP 14 was clustered with Italy strains (ST258-K28BO, ST258-K26BO, and ST512-K30BO) and USA strains. The result was similar to genome tree.

A cgMLST scheme of* K. pneumoniae* was defined using NCBI data in this study. Using* K. pneumoniae* KCTC 2422 strain as reference genome (4,923 genes) and the genome of further seven* K. pneumoniae* strains as query genomes, we defined the standard set of 3,548 genes for the cgMLST scheme.

The resistomes of strain MP14 and other isolates are presented in Figure 1. The strain MP14 possessed the following resistance genes: four *β*-lactamase genes *bla*_KPC-2_, *bla*_SHV-11_, *bla*_TEM-169_ , and *bla*_OXA-9_;* aac(6*′*)-Ib*,* aadA2,* and* aph*(*3*′)*-Ia* as aminoglycoside resistance-encoding genes;* mph(A)* for macrolides;* oqxA* and* oqxB* for quinolone;* catA1 *for phenicol;* sul1* for sulfonamide; and* dfrA12* for trimethoprim. The KPNIH series isolated in the USA and three Italian strains had very similar resistomes to that of strain MP14.

**Figure 4:**
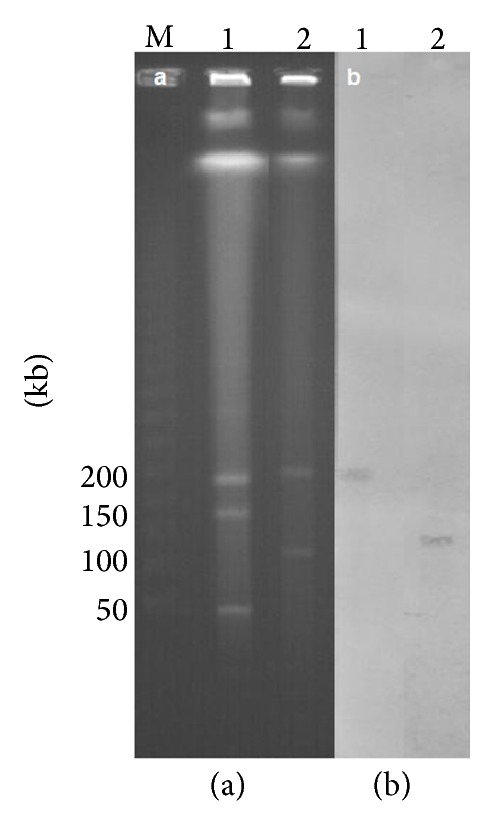
PFGE patterns of S1-digested DNA fragments (a) and Southern blotting with the *bla*_KPC-2_ probe (b). Lane M, lambda ladder (Bio-Rad) as a DNA size marker; lane 1, a clinical isolate of* K. pneumoniae*; lane 2, the strain MP14.

We analyzed the WGS of the related strains using a web tool, PlasmidFinder, for characterization of plasmids [19]. Plasmid-scaffold genes such as* FII(K)*,* FIB(K)*,* FIB(pQil), *and* ColRNAI* were present in the strain MP14, in all isolates of KPNIH strains compared, and in the three Italian strains (Figure 1).* FIB(K)* is known to be associated with plasmid pKPN3 identified in the USA (GenBank accession number CP000648) and plasmid pKPN-IT identified in Italy (JN233704)^19^.* FII(K)* and* FIB(pQil)* are plasmid-scaffold regions of plasmids pKpQil and pKpQil-IT (NC_014016 and JN233705, resp.), which are approximately 115 kb sized plasmids carrying the *bla*_KPC-3_ gene [20]. Based on hybridization of S1-digested DNA fragments of the strain MP14, the *bla*_KPC-2_ probe hybridized with an approximately 120 kb plasmid (Figure 4).

## 4. Discussion 

WGS was carried out for a KPC-2-producing* K. pneumoniae* strain that was isolated in Korea in 2011. Genome sequences of 24 KPC-2-producing* K. pneumoniae* strains (isolated in USA and Italy) similar to Korean strain MP14 were obtained from the NCBI database. Strain MP14 could be compared among strains isolated in different countries or at different times by whole genome sequences and various information of strain could be analyzed. The strain MP14 carried several antimicrobial resistance genes for different antibiotic classes and showed multidrug resistance as expected.

The cgMLST scheme in* K. pneumoniae *isolate was constructed in this study, for the first time. The cgMLST results were comparable to those of the SNPs tree in* K. pneumoniae* isolates (Figure 3). The cgMLST may be a promising tool for standardized, portable, and expandable approach in* K. pneumoniae* isolates although this study has a limitation stemming from its small sample size.

Both SNP and cgMLST based whole genome sequences supported the closer relatedness MP14 strain, KPN NIH strains, and Italy ST strains carrying *bla*_KPC_ genes. These suggested that the transmission of KPC-producing* K. pneumoniae* did not happen by mobile genetic elements but by spread of clone. Although we could not obtain the full sequence of the *bla*_KPC-2_ gene-associated plasmids in the MP14 strain, this strain was found to possess several plasmid-scaffold genes that were previously reported in plasmids pKpQil from Israel and pKpQil-IT from Italy, which carried the *bla*_KPC-2_ genes pKPN3 from the USA and pKPN-IT from Italy. These findings suggest transfer of plasmids carrying the *bla*_KPC_ gene together with the host strains from the USA or Italy to Korea.

## 5. Conclusion 

Whole genome sequencing and its in-depth analysis of the KPC-2-producing MDR* K. pneumoniae* ST258 strain from Korea made it evident that this was strongly related to MDR* K. pneumoniae *strains from USA and Italy. Global spread of KPC-producing* K. pneumoniae *is a worrying phenomenon and close inspection to stop their spread is strongly warranted.

Consider nucleotide sequence accession numbers: the nucleotide sequence data reported in this paper are available in the DDBJ/EMBL/GenBank nucleotide database under accession number ATAK00000000.

## References

[1] Yigit H, Queenan AM, Anderson GJ (2001). Novel carbapenem-hydrolyzing *β*-lactamase, KPC-1, from a carbapenem-resistant strain of *Klebsiella pneumoniae*. *Antimicrobial Agents and Chemotherapy*.

[2] Deshpande LM, Jones RN, Fritsche TR, Sader HS (2006). Occurrence and characterization of carbapenemase-producing enterobacteriaceae: report from the SENTRY Antimicrobial Surveillance Program (2000–2004). *Microbial Drug Resistance*.

[3] Nordmann P, Cuzon G, Naas T (2009). The real threat of *Klebsiella pneumoniae* carbapenemase-producing bacteria. *The Lancet Infectious Diseases*.

[4] Patel G, Huprikar S, Factor SH, Jenkins SG, Calfee DP (2008). Outcomes of carbapenem-resistant *Klebsiella pneumoniae* infection and the impact of antimicrobial and adjunctive therapies. *Infection Control and Hospital Epidemiology*.

[5] Cai JC, Zhou HW, Zhang R, Chen GX (2008). Emergence of *Serratia marcescens, Klebsiella pneumoniae*, and *Escherichia coli* Isolates possessing the plasmid-mediated carbapenem-hydrolyzing beta-lactamase KPC-2 in intensive care units of a Chinese hospital. *Antimicrobial Agents and Chemotherapy*.

[6] Tóth Á, Damjanova I, Puskás E (2010). Emergence of a colistin-resistant KPC-2-producing *Klebsiella pneumoniae* ST258 clone in Hungary. *European Journal of Clinical Microbiology and Infectious Diseases*.

[7] Andrade LN, Curiao T, Ferreira JC (2011). Dissemination of bla_KPC-2_by the spread of *Klebsiella pneumoniae* clonal complex 258 clones (ST258, ST11, ST437) and plasmids (IncFII, IncN, IncL/M) among Enterobacteriaceae species in Brazil. *Antimicrobial Agents and Chemotherapy*.

[8] Bogdanovich T, Adams-Haduch JM, Tian G (2011). Colistin-resistant, *Klebsiella pneumoniae* Carbapenemase (KPC)-producing *Klebsiella pneumoniae* belonging to the international epidemic clone ST258. *Clinical Infectious Diseases*.

[9] Roh KH, Lee CK, Sohn JW, Song W, Yong D, Lee K (2011). Isolation of a *Klebsiella pneumoniae* isolate of sequence type 258 producing KPC-2 carbapenemase in Korea. *The Korean Journal of Laboratory Medicine*.

[10] Hong SK, Yong D, Kim K (2013). First outbreak of KPC-2-producing Klebsiella pneumoniae sequence type 258 in a hospital in South Korea. *Journal of Clinical Microbiology*.

[11] Delcher AL, Bratke KA, Powers EC, Salzberg SL (2007). Identifying bacterial genes and endosymbiont DNA with Glimmer. *Bioinformatics*.

[12] Tatusov RL, Koonin EV, Lipman DJ (1997). A genomic perspective on protein families. *Science*.

[13] Disz T, Akhter S, Cuevas D (2010). Accessing the SEED genome databases via Web services API: tools for programmers. *BMC Bioinformatics*.

[14] Lim Y, Lee Y, Seo Y (2013). Loss of blaVIM-2 and blaIMP-1 during the storage of Gram-negative bacilli, antimicrobial susceptibility of the gene- lost strain, and location of the gene in the cell. *Annals of Clinical Microbiology*.

[15] Kohl TA, Diel R, Harmsen D, Rothgänger J, Meywald Walter K, Merker M (2014). Whole genome based *Mycobacterium tuberculosis* surveillance: a standardized, portable and expandable approach. *Journal of Clinical Microbiology*.

[16] Goris J, Konstantinidis KT, Klappenbach JA, Coenye T, Vandamme P, Tiedje JM (2007). DNA-DNA hybridization values and their relationship to whole-genome sequence similarities. *International Journal of Systematic and Evolutionary Microbiology*.

[17] Snitkin ES, Zelazny AM, Thomas PJ (2012). Tracking a hospital outbreak of carbapenem-resistant *Klebsiella pneumoniae* with whole-genome sequencing. *Science Translational Medicine*.

[18] Comandatore F, Sassera D, Ambretti S (2013). Draft genome sequences of two multidrug resistant *Klebsiella pneumoniae* ST258 isolates resistant to colistin. *Genome Announcements*.

[19] Carattoli A, Zankari E, Garcìa-Fernandez A (2014). PlasmidFinder and pMLST: in silico detection and typing of plasmids. *Antimicrobial Agents and Chemotherapy*.

[20] García-Fernández A, Villa L, Carta C (2012). *Klebsiella pneumoniae* ST258 producing KPC-3 identified in Italy carries novel plasmids and OmpK36/OmpK35 porin variants. *Antimicrobial Agents and Chemotherapy*.

